# Astrocyte reactivity with late-onset cognitive impairment assessed in vivo using ^11^C-BU99008 PET and its relationship with amyloid load

**DOI:** 10.1038/s41380-021-01193-z

**Published:** 2021-07-15

**Authors:** Valeria Calsolaro, Paul M. Matthews, Cornelius K. Donat, Nicholas R. Livingston, Grazia D. Femminella, Sandra Silva Guedes, Jim Myers, Zhen Fan, Robin J. Tyacke, Ashwin V. Venkataraman, Robert Perneczky, Roger Gunn, Eugenii A. Rabiner, Steve Gentleman, Christine A. Parker, Philip S. Murphy, Paul B. Wren, Rainer Hinz, Magdalena Sastre, David J. Nutt, Paul Edison

**Affiliations:** 1 Department of Brain Sciences, Imperial College London, London, UK; 2 UK Dementia Research Institute, Imperial College London, London, UK; 3 Centre for Blast Injury Studies, Imperial College London, London, UK; 4 Department of Psychiatry and Psychotherapy, University Hospital, LMU Munich, Munich, Germany; 5 German Centre for Neurodegenerative Disorders (DZNE), Munich, Germany; 6 Munich Cluster for Systems Neurology (SyNergy), Munich, Germany; 7 Ageing Epidemiology Research Unit (AGE), School of Public Health, Imperial College London, London, UK; 8 Invicro, London, UK; 9 King’s College London, London, UK; 10 GlaxoSmithKline, Stevenage, UK; 11 Wolfson Molecular Imaging Centre, University of Manchester, Manchester, UK; 12 Cardiff University, Cardiff, Wales, United Kingdom

**Keywords:** Prognostic markers, Neuroscience

## Abstract

^11^C-BU99008 is a novel positron emission tomography (PET) tracer that enables selective imaging of astrocyte reactivity in vivo. To explore astrocyte reactivity associated with Alzheimer’s disease, 11 older, cognitively impaired (CI) subjects and 9 age-matched healthy controls (HC) underwent 3T magnetic resonance imaging (MRI), ^18^F-florbetaben and ^11^C-BU99008 PET. The 8 amyloid (Aβ)-positive CI subjects had higher ^11^C-BU99008 uptake relative to HC across the whole brain, but particularly in frontal, temporal, medial temporal and occipital lobes. Biological parametric mapping demonstrated a positive voxel-wise neuroanatomical correlation between ^11^C-BU99008 and ^18^F-florbetaben. Autoradiography using ^3^H-BU99008 with *post-mortem* Alzheimer’s brains confirmed through visual assessment that increased ^3^H-BU99008 binding localised with the astrocyte protein glial fibrillary acid protein and was not displaced by PiB or florbetaben. This proof-of-concept study provides direct evidence that ^11^C-BU99008 can measure in vivo astrocyte reactivity in people with late-life cognitive impairment and Alzheimer’s disease. Our results confirm that increased astrocyte reactivity is found particularly in cortical regions with high Aβ load. Future studies now can explore how clinical expression of disease varies with astrocyte reactivity.

## Introduction

Astrocyte reactivity is a prominent feature of the neuropathology of Alzheimer’s disease (AD), but the extent to which it is a consequence or a contributing factor for the formation of amyloid (Aβ) and tau aggregation remains uncertain [1]. Astrocytes have a wide range of roles in the central nervous system [2, 3], and astrocyte reactivity can increase expression of inflammatory mediators, reactive oxygen species and Aβ deposition in mouse models [2]. It has been suggested that astrocyte reactivity could be a tissue response to Aβ deposition and may have protective roles by phagocytosing and degrading Aβ [4, 5]. Human data concerning astrocyte reactivity in AD is largely limited to that from neuropathology *post-mortem*. Whilst some methodologies exist to measure astrocyte reactivity in living AD patients, such as CSF [6, 7], blood [8] and positron emission tomography (PET) [9] biomarkers, there are issues surrounding their sensitivity and specificity [10]. Non- or minimally invasive methods for monitoring astrocyte reactivity in patients with late-life cognitive impairment would provide a powerful tool for testing their contributions to disease progression. Currently, the only available PET tracer which can measure astrocyte reactivity in vivo is ^11^C-deuterium-L-deprenyl (^11^C-DED) [10, 11]. However, this tracer binds to monoamine oxidase-B, which is not exclusively expressed in astrocytes and is not elevated in late disease stages when Aβ load is high [12]. Additional radioligands are needed to confidently image astrocyte reactivity and its relations to Aβ load and clinical symptoms.

Non-adrenergic imidazoline binding in astrocytes is of the I_2_ sub-class with the putative receptor localised mainly in the mitochondria [13]. *Post-mortem* neuropathology described upregulation of I_2_-BS sites with aging [14] and in AD [15]. In order to make corresponding observations in living subjects, a novel approach using a PET tracer, ^11^C-BU99008, which binds specifically to I_2_-BS, has been developed [13, 16–18]. ^11^C-BU99008 showed good brain penetration in rodents [19], pigs [20] and non-human primates [20–22]. Subsequent studies showed favourable biodistribution [23] and dosimetry profiles in humans [24].

This pilot study was designed to test, for the first time, whether the uptake of ^11^C-BU99008 is increased in older cognitively impaired (CI) subjects relative to approximately age-matched healthy controls (HC). We then characterised the distribution of uptake of ^11^C-BU99008 across the brain. Finally, we explored the neuroanatomical associations between ^11^C-BU99008 uptake and Aβ deposition assessed using ^18^F-florbetaben which will allow us to test in the future the more specific hypothesis that astrocyte reactivity is an early and dynamic response to the neuropathology of AD.

## Materials and methods

### Subjects

Eleven subjects with CI who were clinically diagnosed as having probable AD dementia or mild cognitive impairment (MCI) due to AD [25, 26] (5 MCI, 6 AD; Mini-Mental State Examination score (MMSE) mean ± SD = 22.6 ± 4.1) and met the additional criteria below and 9 approximately age-matched HC without a history of brain disease or contraindications to  magnetic resonance imaging (MRI; MMSE mean ± SD = 29.1 ± 1.27) were included in the study (Table 1). All 20 subjects were recruited from memory clinics, advertisements and research registries. Additional inclusion/exclusion criteria for the CI subjects were that they needed to be able to give informed consent, have at least 8 years of education, an MMSE score ≥17 when enroled in the study and not have prior evidence of significant vascular or small vessel disease on MRI. All subjects underwent medical, neurological and detailed cognitive assessments using the Repeatable Battery for the Assessment of Neuropsychological Status (RBANS). Ethical approval for this study was granted by the local and regional Research Ethics Committee. Approval to administer radiotracers was given by the Administration of Radioactive Substances Advisory Committee UK. Written informed consent was obtained from all participants. The human biological samples sourced from the subjects participating in this study were obtained ethically and their research use was in accordance with the terms of the informed consents.

**Table 1:** Demographics and cognitive test scores (cognitively impaired subjects vs healthy control subjects).

	*N*	Sex (M:F)	Age in Years Mean (±SD)	Global Aβ PET SUVr Mean (±SD)	MMSE Mean (±SD)	Immediate Memory Mean (±SD)	Visuospatial/Constructional Mean (±SD)	Language Mean (±SD)	Attention Mean (±SD)	Delayed Memory Mean (±SD)
HC	9	8:3	69.8 (±8.5)	1.21 (±0.06)	29.1 (±1.27)	115.4 (±11)	100.5 (±16)	100.8 (±6.34)	110 (±12.7)	105 (±7.8)
CI Subjects	11 (8 Aβ+, 3 Aβ−)	5:4	74 (±4.5)	1.55 (±0.29)	22.6 (±4.1)	65.5 (±20)	86 (±20.6)	81.5 (±15.4)	87.5 (±19)	61.5 (±17.3)
*P* values	N/A	N/A	0.919	0.004*	0.001*	<0.001*	0.172	0.003*	0.012	<0.001*

^*^p < 0.01 between cognitively impaired and healthy control subjects.

*Aβ+* amyloid positive, *Aβ−* amyloid negative, *F* female, *M* male, *MMSE* mini-mental state examination, *SD* standard deviation, *SUVr* standard uptake value ratio.

### MRI

All subjects underwent MRI with either a 3 Tesla Magnetom Trio or Verio (Siemens Healthcare Sector, Erlangen, Germany) with a 32-receiver channel head matrix coil. A sagittal T1-weighted MPRAGE was acquired with TR = 2400 ms, TE = 3.06 ms, flip angle = 9°, TI = 900 ms, matrix = [256 × 246], a 1 mm isotropic voxel size, anteroposterior phase encoding direction, IPAT factor 2 and a symmetric echo.

### PET

#### ^18^F-florbetaben PET

All subjects underwent ^18^F-florbetaben PET scanning to assess Aβ plaque deposition in the brain. Subjects received a mean of 236.4 (±6.8) MBq ^18^F-florbetaben as a single intravenous bolus. PET acquisition was commenced 90 min after ^18^F-florbetaben administration and subjects were scanned for 30 min. ^18^F-florbetaben uptake was evaluated using the standardised uptake value ratio (SUVR) of the cerebral cortex with cerebellar grey matter (GM) as reference [27]. For this, regional uptake in cerebellum was calculated in Analyze 11.0 (developed by the Biomedical Imaging Resource at the Mayo Clinic). The 90–120 min ^18^F-florbetaben ratio images were created by dividing the cerebral cortical ^18^F-florbetaben image by the uptake value of cerebellar GM. Aβ positivity was defined by using a whole-brain uptake cut-off of 1.43 [27].

#### ^11^C-BU99008 PET

^11^C-BU99008 was synthesised at the Invicro Centre for Imaging Sciences in London and imaging was performed at the same centre with a Siemens Truepoint PET/CT. An initial CT scan was acquired for attenuation correction of the PET images. A mean activity of 330 (±30) MBq ^11^C-BU99008 in 20 ml normal saline was injected into the antecubital vein. Dynamic emission ^11^C-BU99008 PET images were then acquired over 120 min and rebinned in 29 time frames: 8 × 15, 3 × 60, 5 × 120, 5 × 300, and 8 × 600 s. Non-attenuation corrected ^11^C-BU99008 PET images were also created for motion correction in MIAKAT^TM^ (www.miakat.org). All subjects had a radial arterial cannula inserted, and arterial blood was sampled continuously for the first 15 min. Twelve additional samples were taken at 5, 10, 15, 20, 25, 30, 40, 50, 60, 70, 80, and 100 min after injection. The radioactivity in the whole blood and plasma was measured using a gamma-counter for each sample. Metabolism of ^11^C-BU99008 was evaluated by reverse-phase high-performance liquid chromatography, which determined the relative proportions of parent tracer and metabolites in the blood. In this study, we used MIAKAT^TM^ to perform the spatial normalisation and compartmental modelling. In order to generate the parent plasma input function, the radioactivity in the whole blood in the first 15 min was estimated by calibrating the first 15 min of continuous whole blood data to the gamma-counter measurements. The ratio of radioactivity concentration in the plasma to blood measurements, and the fraction of parent compound in the blood, were both fitted with sigmoid curves as described previously [23]. These models were used to correct the whole blood data, and the resulting input function fitted by a tri-exponential model [28].

### Region of interest (ROI) analysis

To create individual object maps, each structural MRI was segmented into GM, white matter (WM) and CSF, and the GM images were thresholded at 50% probability. The structural T1 volumetric MRI, GM MRI and 3D PET data were co-registered into a mutual space. The deformation fields to transform the individual’s MRI to the standard MNI template were calculated, and the inverse of these parameters was applied to the CIC atlas to create an atlas in individual subject’s native PET space. This was then used to generate individualised ROIs for each participant. VT was calculated using 2TCM for frontal, temporal, medial temporal, parietal and occipital lobe, as well as the cerebellum, composite cortex and other subcortical regions in PET space. Time–activity curves for selected ROIs were generated by sampling the radioactivity concentration of the motion-corrected dynamic PET in native space. Regional estimates of total volumes of distribution (*V*_T_) were obtained with the four-parameter reversible two-tissue compartmental model (2TCM4k), as previously described [24, 28].

These analyses were extended by applying spectral analysis to ^11^C-BU99008 dynamic images to generate parametric maps [29, 30]. The Impulse Response Function at 120 min (IRF-120) was chosen to create ^11^C-BU99008 parametric maps using Modelling, Input Functions and Compartmental Kinetics Parametric Map (MICK-PM) software (available on request from RH, Wolfson Molecular Imaging Centre, University of Manchester, Manchester, UK). After creating IRF-120 parametric maps for each subject using spectral analysis, parametric maps were co-registered to the individual’s T1 volumetric MRI. The T1 volumetric MRI and GM MRI were normalised to the MNI template using Analyze 11.0, and these deformation fields were applied to the 3D PET data to transform them into MNI space. The normalised parametric maps were then sampled to generate ROI results. For voxel-wise statistical parametric mapping (SPM) and biological parametric mapping analysis in SPM, all parametric images were smoothed using a 6 × 6 × 6 mm^3^ filter in SPM8.

### Statistical parametric mapping (SPM) analysis

A limitation of the ROI-based analyses above is that they describe average tracer uptake across pre-defined anatomical regions. To better characterise the neuroanatomical distributions of differences in uptake between groups, we also performed voxel-level SPM analyses. ^11^C-BU99008 uptake was estimated using IRF-120 in CI subjects against HC using a two-sample Student’s *t* test (two-tailed, threshold *p* < 0.05; cluster extent threshold 50 voxels). To perform single-subject analyses, *t t*ests in SPM8 were performed, comparing each CI subject’s normalised and smoothed (6 × 6 × 6 mm in SPM8) image against the group of HC subjects.

### Biological parametric mapping (BPM) correlation analysis

In order to evaluate the neuroanatomical relationships between Aβ and ^11^C-BU99008 binding, *Z*-score maps for each PET modality were created, which represented ^11^C-BU99008 binding and Aβ deposition compared with the HC’ mean and standard deviation on an individual level on a voxel-wise basis using the following formulae:

$$	{\mathrm{Zmap}}\, {\mathrm{of}}\,^{11}{\mathrm{C}}{\hbox{-}} {\mathrm{BU99008}} \\ 	=\frac{{\mathrm{cognitively}}\,{\mathrm{impaired}}\,{\mathrm{subject}}\,^{11}{\mathrm{C}}{\hbox{-}}{{\mathrm{BU99008}} - {\mathrm{mean}}\,{\mathrm{of}}\,{\mathrm{healthy}}\,{\mathrm{controls}}\,^{11}{\mathrm{C}}{\hbox{-}}{\mathrm{BU99008}}}}{{\mathrm{SD}}\,{\mathrm{of}}\,{\mathrm{healthy}}\,{\mathrm{controls}}\, ^{11}{\mathrm{C}}{\hbox{-}}{\mathrm{BU99008}}}$$

$$	{\mathrm{Zmap}}\, {\mathrm{of}}\,^{18}{\mathrm{F}}{\hbox{-}} {\mathrm{florbetaben}} \\ 	=\frac{{\mathrm{cognitively}}\,{\mathrm{impaired}}\,{\mathrm{subject}}\,^{18}{\mathrm{F}}{\hbox{-}}{{\mathrm{florbetaben}} - {\mathrm{mean}}\,{\mathrm{of}}\,{\mathrm{healthy}}\,{\mathrm{controls}}\,^{18}{\mathrm{F}}{\hbox{-}}{\mathrm{florbetaben}}}}{{\mathrm{SD}}\,{\mathrm{of}}\,{\mathrm{healthy}}\,{\mathrm{controls}}\, ^{18}{\mathrm{F}}{\hbox{-}}{\mathrm{florbetaben}}}$$

Then, a voxel-level correlation between the *Z*-score maps for ^11^C-BU99008 uptake and Aβ deposition was estimated using BPM [31]. The BPM toolbox runs on the MATLAB platform and is integrated into the SPM software package [32]. The BPM toolbox performs multimodal correlations and multiple regression at a voxel-level using the general linear model to provide sophisticated voxel-wise correlation. *Z*-score maps were created (instead of using the raw image) in order to remove any non-specific uptake of ^18^F-florbetaben (such as WM uptake) which occurs in healthy populations. Thus, when the correlation is performed, we will only be correlating residual pathology which is disease-related, and therefore reduce the likelihood of artificial correlations not related to disease pathology. In addition, Z-mapping is the recommended way of performing BPM correlation [31]. Correlations for all CI subjects and for the subgroup of Aβ-positive subjects were evaluated separately. Results are displayed with a cluster threshold of *p* < 0.05 and extent threshold of 50 voxels.

### In vitro autoradiography and immunostaining

^11^C-BU99008 PET has not been validated previously for AD. We performed autoradiographic studies of ^3^H-BU99008 binding to *post-mortem* AD and non-disease control brains of similar age to test for specific (displaceable) cortical GM binding, greater uptake with Alzheimer’s pathology and co-localisation with glial fibrillary acid protein (GFAP), an astrocyte marker. Details are described in Supplementary Methods. Briefly, to address specificity of binding, human frontal cortical tissue sections (12–14 µm thick) from *post-mortem* brains with Alzheimer pathology were incubated with 2–3.5 nMol/L ^3^H-BU99008 for 60 min in assay buffer, with or without an excess of unlabelled BU224 (a ligand that competes for the same I_2_B site as BU99008). Frontal cortex tissue was used as this is one of the main area’s that Aβ deposition occurs first [33]. Additional experiments were performed to enhance confidence in target specificity of ^3^H-BU99008 in cortical sections with high Aβ plaque densities by testing for competition with excesses of either unlabelled Pittsburgh Compound-B (PiB) or florbetaben. Finally, quantitative assessments of ex vivo ^3^H-BU99008 binding in AD and control tissue were performed, in conjunction with immunohistochemical staining of contiguous sections for GFAP. Following washing, slides were exposed along with radioactive standards and imaged by phosphorimaging. ROIs were manually drawn of the whole section, GM and WM, and optical density per mm^2^ was converted to fmol radioligand/mg wet tissue equivalent. Localisation of increased ^3^H-BU99008 binding and GFAP staining on adjacent sections were compared visually.

## Results

### ^18^F-florbetaben ROI analysis

All of the HC were Aβ-negative. 8/11 CI subjects were Aβ-positive (3 MCI, 5 AD) and 3/11 were Aβ-negative (2 MCI, 1 AD). Ages of Aβ-positive (mean age ±SD, 76 ± 4 years old) and Aβ-negative (71 ± 4 years old) CI subjects were similar to the HC group (70 ± 8 years old; See Table 1). Global tracer uptake (mean uptake ±SD) was 1.55 ± 0.29 for CI subjects and 1.21 ± 0.06 for HC. Shapiro-Wilk’s tests confirmed data of global tracer uptake were normally distributed for CI subjects (W(11) = 0.948, *p* = 0.616) and HC (W(9) = 0.956, *p* = 0.751).

### ^11^C-BU99008 ROI analysis

**Fig. 1:**
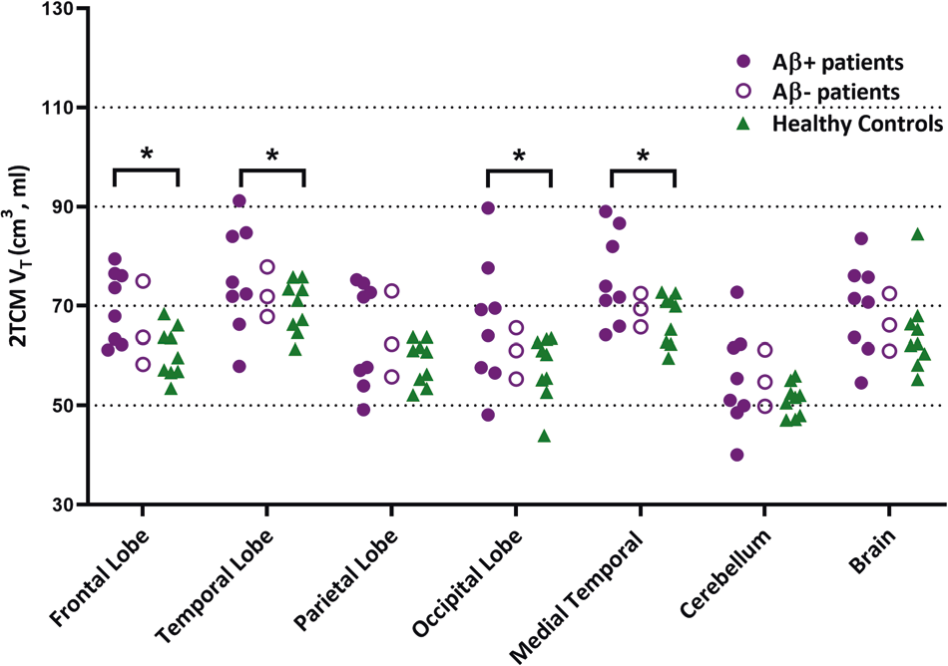
^11^C-BU99008 2TCM V_T_ in different cortical regions. Dot plot demonstrating the regional ^11^C-BU99008 2TCM V_T_ in Aβ-positive CI subjects (purple filled circle), Aβ-negative CI subjects (purple open circle) and HC (green triangle). “Brain” refers to the composite cortex, combining all the major cortical regions. *denotes *p* < 0.05, uncorrected.

Results for the ROI analysis of ^11^C-BU99008 uptake are shown in Fig. 1. Global tracer uptake (mean uptake ±SD) was 82.7 ± 11.5 for CI subjects and 77.7 ± 7.7 for HC. Shapiro–Wilk’s tests confirmed data of global tracer uptake were normally distributed for CI subjects (W(11) = 0.942, *p* = 0.546) and HC (W(9) = 0.869, *p* = 0.095). Contrast of major brain ROI V_T_ generated from a 2TCM showed increased uptake in the CI subjects compared to HC in the frontal (17%, *p* = 0.007, uncorrected, two-tailed Student’s *t* test) cortex. Aβ-positive CI subjects had higher ^11^C-BU99008 uptake in the frontal (21%, *p* = 0.004), temporal (15%, *p* = 0.034), medial temporal (18%, *p* = 0.015) and occipital (24%, *p* = 0.026) lobes.

We extended the analysis using model-free IRF parametric maps (Supplementary Fig. 1) for CI subjects who were further stratified *post hoc* based on Aβ status. Contrasting the Aβ-positive CI subjects with HC, IRF-120 demonstrated increased uptake over the whole brain (22% increase, *p* = 0.008, uncorrected) and in frontal (20%, *p* = 0.03), temporal (19%, *p* = 0.01), medial temporal (14%, *p* = 0.022), parietal (25%, *p* = 0.003) and occipital (26%, *p* = 0.006) lobes.

We performed Pearson correlations to investigate the relationship between IRF-120 and 2TCM images in each participant (Supplementary Fig. 2). We found very strong correlations across each of the four main lobes, as well as in smaller ROIs, such as the hippocampus (*r* = 0.86, *p* < 0.001) and amygdala (*r* = 0.91, *p* < 0.001), despite increased noise in these regions. One HC (53yo female) was located as an outlier for the temporal lobe, perhaps due to her younger age. Compared to other HCs, this individual had a somewhat higher MMSE (30, mean of HC = 29.25) and high RBANS (120, mean of HC = 105) score, was Aβ-negative according to their ^18^F-florbetaben scan and had a hippocampal volume of 3607 mm^3^ (mean of HC = 3792 mm^3^).

### BPM correlations between ^11^C-BU99008 binding and Aβ deposition

**Fig. 2:**
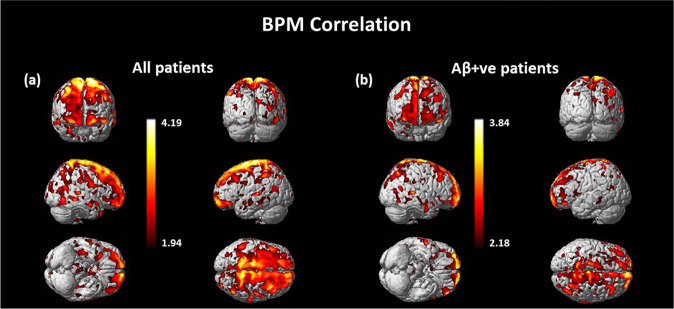
BPM correlation between ^11^C-BU99008 and Aβ deposition. BPM correlation between ^11^C-BU99008 and ^18^F-florbetaben binding in (**a**) all CI subjects and in (**b**) Aβ-positive CI subjects at a cluster threshold of *p* < 0.05 with an extent threshold of 50 voxels. These BPM are T maps describing the strength of the voxel-wise correlations between binding of the two radioligands represented in a common brain space.

Voxel-based BPM analysis demonstrated a strong positive correlation between cortical ^11^C-BU99008 and ^18^F-florbetaben binding in Aβ-positive subjects (Fig. 2). A similar relationship was found with a BPM analysis across the whole group studied (Fig. 2).

### SPM analysis

**Fig. 3:**
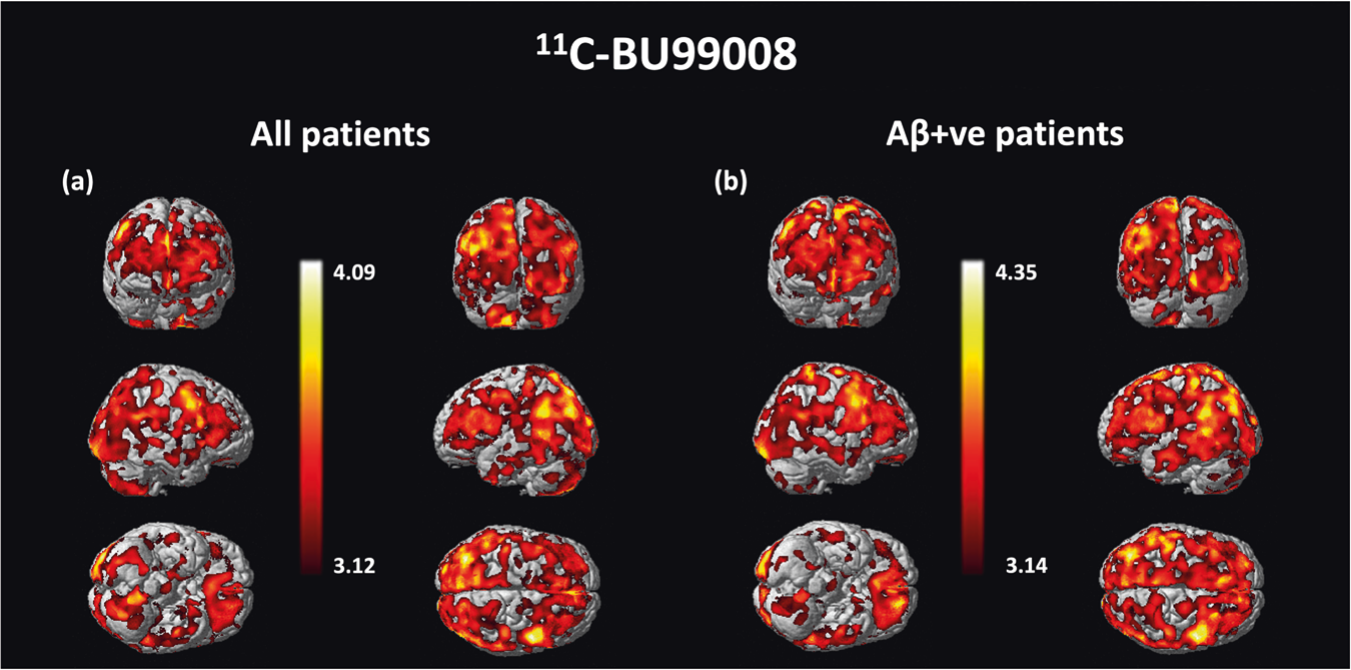
SPM analysis of ^11^C-BU99008. SPM analysis of significant increased ^11^C-BU99008 uptake as a group for (**a**) all CI subjects and (**b**) Aβ-positive CI subjects compared with HC, using a cluster threshold of *p* < 0.05 and with extent threshold of 50 voxels. The Colour bar indicates the *Z*-score.

Voxel-wise SPM of the whole CI group and for those who were Aβ-positive demonstrated increased ^11^C-BU99008 uptake in clusters of voxels predominantly in the frontal, parietal, occipital and temporal cortex and the cerebellum, consistent with the ROI and BPM analyses (Fig. 3). Whole-brain voxel-level analyses of the individual CI subjects showed significantly increased uptake in clusters of cortical voxels in 8/11 of CI (6/8 Aβ-positive; 2/3 Aβ-negative) relative to HC (Supplementary Fig. 3).

### In vitro autoradiography and immunohistochemistry

**Fig. 4:**
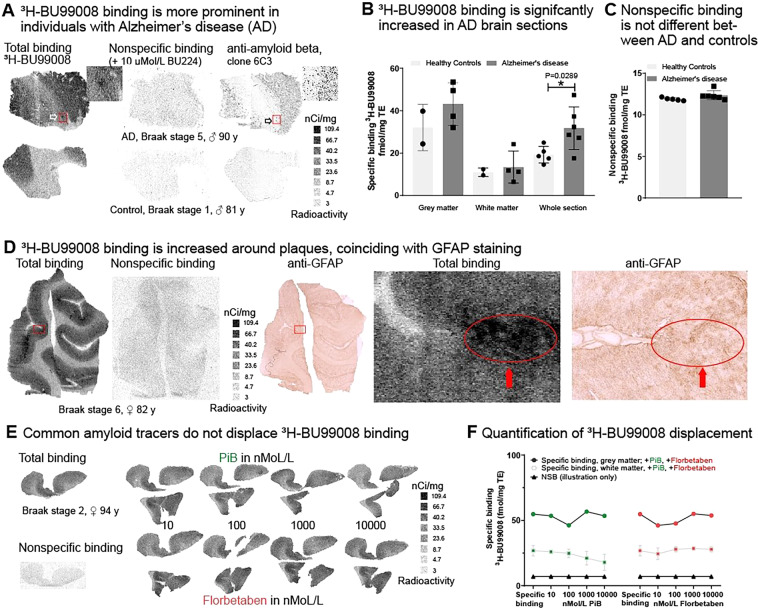
In vitro autoradiography of ^3^H-BU99008 in brain tissue from people with AD and controls. **A** Greater ^3^H-BU99008 binding around Aβ plaques in AD brain tissue sections. Representative autoradiographs showing ^3^H-BU99008 total (left panel) and non-specific binding (centre panel; determined with 10 µM BU224) in 12 µm frontal cortex sections of the human brain (AD and age-matched controls). Standards (ARC 123B) represent a linear range of radioactivity. Immunohistochemistry for total Aβ (1 µg/mL pan-anti-Aβ, MOAB-2, clone 6C3, right panel) shows spatial distribution of plaques. White arrows point to high-intensity binding of ^3^H-BU99008 in the vicinity of plaques (black arrows), as magnified inserts show. **B** ^3^H-BU99008 binding is increased in AD brain sections relative to control brain. Comparative quantitative analysis of specific binding of ^3^H-BU99008 in grey and white matter (when identifiable) or total section of 12 µm frontal cortex sections (AD with Braak stages 5–6 and controls). Binding of ^3^H-BU99008 in the total sections from AD cases is significantly higher (unpaired t-test) than that in controls. Data is mean (±SD) from triplicates; AD: *n* = 6, Control: *n* = 5. **C** Non-specific binding is not different between AD and control brain sections. Quantitative analysis of non-specific binding, showing no difference between brain sections from AD and controls. Increased specific binding in AD (**A**/**B**) sections relative to controls therefore is not caused by differences in non-specific binding. **D** ^3^H-BU99008 binding colocalised with GFAP staining. Autoradiograph showing ^3^H-BU99008 total binding (left panel) and non-specific binding (centre panel; determined with 10 µM BU224) in 14 µm frontal cortex sections of the human brain (Braak stage 6). Standards (ARC 123B) represent a linear range of radioactivity. Immunohistochemistry for GFAP (right panel) shows spatial distribution of an astrocytic marker. Magnifications of ^3^H-BU99008 binding and GFAP staining are shown in the panels. Solid red arrows point to high-intensity ^3^H-BU99008 binding and corresponding areas of GFAP staining. **E**, **F** Common Aβ tracers do not displace ^3^H-BU99008 binding. Autoradiograph showing ^3^H-BU99008 total binding (left upper panel) and non-specific binding (left lower panel; determined with 10 µM BU224) in 14 µm frontal cortex sections of the human brain (Braak stage 2). Standards (ARC 123B) represent a linear range of radioactivity. Upper/lower right row shows binding of ^3^H-BU99008 in the presence of two commonly unlabelled PiB and Florbetaben, two commonly used Aβ tracers in ascending concentrations, showing no displacement. Quantitative analysis of specific binding (**E**) in the grey and white matter and in the presence of unlabelled PiB and Florbetaben (10–10000 nMol/L) in 14 µm frontal cortex sections (Braak stage 2). Quantification indicates that high-intensity ^3^H-BU99008 accumulation around plaques is likely not caused by binding to common Aβ-binding sites.

We performed in vitro ^3^H-BU99008-binding studies using *post-mortem* frozen sections of frontal cortex from AD patients and from HC to validate the in vivo ^11^C-BU99008 PET observations. Total binding of ^3^H-BU99008 was higher in cortical GM than WM, with a clear distinction between binding in the two-tissue compartments apparent visually (Fig. 4A, B). Quantitatively, higher specific binding was observed across whole sections from AD brains relative to the HC tissue (t(9) = 2.596, *p* = 0.0289; Fig. 4B). This binding was consistent with a high-affinity binding site and not due to difference in non-specific binding (Fig. 4C). ^3^H-BU99008 binding was displaceable by excess unlabelled BU224, which binds to the same I2B target (non-specific binding, Fig. 4A, D). Immunostaining in sections adjacent to ^3^H-BU99008 binding showed a good spatial overlap of higher ^3^H-BU99008 binding with anti-GFAP staining for astrocytes (Fig. 4D, red circle). There was no evidence for “off-target” binding of ^3^H-BU99008 to Aβ plaques: PiB or florbetaben in concentrations spanning one order of magnitude (10 nMol/L–10 µMol/L) failed to displace ^3^H-BU99008 binding in *post-mortem* frontal cortex sections from AD patients with Braak stages 2–5 (Fig. 4D, E).

## Discussion

This is the first study evaluating ^11^C-BU99008 PET as a tool for imaging astrocyte reactivity in the brains of subjects with late-life cognitive impairment of the Alzheimer-type. We demonstrated an increase in ^11^C-BU99008 uptake in Aβ-positive MCI or AD subjects using ROI and voxel-wise analyses. In addition, we provided evidence for a correlation between ^11^C-BU99008 and ^18^F-florbetaben uptake in these subjects as a group, consistent with the expected association of astrocyte reactivity with Aβ plaques observed neuropathologically in brains from people with AD. While preliminary, our single-subject analyses suggested that PET Aβ-positivity is neither necessary nor sufficient for elevated local ^11^C-BU99008 uptake (and, by implication, astrocyte reactivity) in people with CI. Finally, we used in vitro autoradiography to confirm increased cortical ^3^H-BU99008 binding in tissue from AD relative to non-disease control brains, the co-localisation of ^3^H-BU99008 binding with staining for GFAP, a marker of reactive astrocytes, and the specificity of binding.

^11^C-BU99008 is a novel PET tracer targeting I_2_-BS. It is hypothesised that I_2_-BS are expressed predominantly in astrocytes, in which it is predominantly localised to the mitochondria [15, 34, 35]. However, despite the pharmacological specificity of binding, no single receptor site has been characterised to date [35]. Preclinical PET evaluations of ^11^C-BU99008 in rhesus monkey brains demonstrated selective uptake (globus pallidus>cortex>cerebellum) in GM consistent with in vitro localisation of I_2_-binding sites by autoradiography and similar to results obtained earlier in porcine brains [20]. A subsequent first-in-human study recently published by our group provided further evidence that ^11^C-BU99008 has high specificity and selectivity for I_2_-BS and validated a two tissue compartment model for analysis of the data [28]. ^11^C-BU99008 has favourable dosimetry and thus appears to be safe for serial administrations in humans [24]. Based on this and the in vitro cellular localisation data, we believe that ^11^C-BU99008 is a potential new tracer for assessment of the trajectory of in vivo astrocyte reactivity with Alzheimer’s and other late life neurodegenerative diseases.

The potential utility of the tracer is suggested by considerable independent neuropathological data that has defined an association between astrocyte reactivity and Aβ deposition in the brain. Studies of both animal models and human brains *post-mortem* demonstrate astrocyte reactivity near Aβ plaques [36, 37]. Consistent with this, in our single-subject SPM analysis, we demonstrated that there were significant clusters of greater ^11^C-BU99008 uptake in 6/8 of the Aβ-positive CI subjects relative to HC. The association between Aβ and astrocyte reactivity is believed to reflect mechanistic links between the two phenomena in a cascade of biochemical events contributing to neurodegeneration. Astrocytes are important mediators of Aβ-induced neurotoxicity and tau phosphorylation in primary culture, e.g., through increased expression of neurotoxic substances such as nitric oxide and TNFα [38]. Astrocytes also play a critical role in regulating brain glucose metabolism homoeostasis [39] and contribute to ^18^F-FDG uptake in the brain [40]. Consequently, a proinflammatory state leading to astrocyte dystrophy will reduce astrocytic glycolytic capacity and, in consequence, neuronal metabolism, potentially leading to neuronal death [41–43]. Therefore, due to the role of astrocyte reactivity on Aβ, tau and glucose metabolism, astrocyte reactivity can lead to atrophy and neurodegeneration and will have a significant impact on the A/T/N framework which should be considered further [44, 45].

We validated our in vivo findings through autoradiography, as ^3^H-BU99008 colocalised with GFAP staining. GFAP is significantly upregulated during astrocyte reactivity [46] as the protein is necessary to build intermediate filaments [47] required for the biological process of hypertrophy that is characteristic of astrocyte reactivity [48]. Consequently, GFAP knockout in an AD mouse model inhibits the development of hypertrophic astrocytes [4]. GFAP also appears to be upregulated in a range of astrocyte reactivity states [49], even in the absence of cell proliferation [50]. Therefore, GFAP is a widely used marker of astrocyte reactivity, and is being investigated as a CSF [51] and blood [52] biomarker for astrocyte reactivity in AD. Ishiki et al. [51] found that whilst CSF GFAP was significantly higher in AD patients compared with controls, CSF GFAP findings were similar with other types of dementia such as dementia with Lewy Bodies and frontotemporal lobar degeneration. This is proposed to be due to a common underlying mechanism of neuroinflammation, and thus suggests CSF GFAP is not suitable for distinguishing between neurodegenerative diseases. We also found that ^3^H-BU99008 colocalised with Aβ plaques and was not displaced by PiB/florbetaben, confirming the tracer was not simply binding to Aβ. The antibody we used to identify amyloid specifically recognises Aβ in plaques (as well as oligomers and fibres). Alternative intercalating markers such as Thioflavin T recognise all aggregated proteins, including tau, that share Aβ structures. Other studies have also shown that GFAP co-localises with Aβ [53–55], and that GFAP can attenuate Aβ load [4, 56], suggesting astrocyte reactivity can occur in response to Aβ pathology.

However, we also observed increased ^11^C-BU99008 uptake in 2/3 of the Aβ-negative CI subjects, suggesting that Aβ plaques detected by ^18^F-florbetaben PET are not necessary for astrocyte reactivity. We have previously demonstrated similar findings with a tracer of microglial activation showing increased uptake in areas with low Aβ load [57]. There is an emerging view that astrocyte reactivity occurs early on in the AD trajectory, and is supported by findings of increased ^11^C-DED binding in autosomal dominant AD patients [58] and Aβ-positive MCI subjects [59]. Importantly, astrocyte reactivity can accelerate Aβ production with increased expression of three elements central to Aβ production: Aβ precursor protein, β-secretase and γ-secretase [60]. The lysis of dead phagocytic astrocytes that have engulfed Aβ peptides may even contribute to the genesis of the consolidated Aβ plaques detected by PET Aβ imaging in vivo [36]. It should also be noted that astrocyte reactivity can occur in other neurodegenerative diseases without Aβ deposition, highlighting that there are multiple triggers [61, 62]. Recent papers have provided in vivo imaging evidence for astrocyte reactivity in Parkinson’s disease, based both on observations of increased ^11^C-BU99008 and ^11^C-DED uptake in the brain [62, 63]. Our observations, these, and related studies thus all emphasise that, while increased ^11^C-BU99008 PET signal may be able to be used as a biomarker for astrocyte reactivity, it is not specific to AD, and could be used to evaluate astrocyte reactivity in different neurodegenerative diseases.

It is more difficult to interpret the lack of any voxel-wise clusters showing significantly increased ^11^C-BU99008 binding relative to HC for the Aβ-positive subject 7 and the relatively small, sparsely distributed clusters found for Aβ-positive subject 5 (Supplementary Fig. 1). Both of these subjects had cognitive and imaging findings (low MMSE scores, high ^18^F-florbetaben uptake and small hippocampal volumes) consistent with inclusion criteria and a clinical diagnosis of probable AD. The relative absence of astrocyte reactivity detected by ^11^C-BU99008 around advanced Aβ plaques could be a marker of a shift in the reactive astrocyte phenotype. It is important to remember that ^11^C-BU99008 binds to I_2_-BS receptor sites as an index of astrocyte reactivity, and thus not all types of astrocyte reactivity will be detected. For example, differences in the molecular phenotypes of reactive astrocytes could lead to relatively lower I_2_B expression in a subset, although earlier unselective biochemical pathology did not suggest much heterogeneity in relative levels of expression between brains studied *post-mortem* [15]. However, the authors noted that GFAP was increased more (88%) than imidazoline receptor proteins (36%) in AD brains, suggesting an astrocyte phenotype which lacks I_2_-BS receptor sites and thus will not be detected by ^11^C-BU99008. Additional ^11^C-BU99008 PET studies of well characterised subjects, as well as ^3^H-BU99008 autoradiography of *post-mortem* brain tissue, are needed to further test and then, if confirmed, explore these phenomena.

## Conclusion

In this proof-of-principle study using the novel PET tracer ^11^C-BU99008, we demonstrated an increase in ^11^C-BU99008 uptake in CI subjects with MCI or probable AD compared with HC. We found that radioligand uptake was associated with Aβ deposition at voxel level, consistent with a potential mechanistic link between Aβ deposition and astrocyte reactivity. The observation that, on an individual subject level, a positive ^18^F-florbetaben PET signal is neither a necessary nor sufficient condition for the increased ^11^C-BU99008 PET signal is most consistent with a model in which astrocyte reactivity can precede significant Aβ plaque deposition and possibly contribute to their formation. While this was only a pilot study, it suggests the potential for using ^11^C-BU99008 for longitudinal study of relationships between astrocyte reactivity and other neuropathological and clinical features in vivo and for exploring the impact of therapeutic approaches targeting astrocyte reactivity.

### Assumptions for power calculation

We assumed that variance in the ROI-based measures may be as much as 15% in the HCs, but, in CI subjects, who may show additional variance related to disease, this will increase to ~30%. Using these assumptions, we calculated the following population sizes to achieve a power of 80% to detect an increase in the subjects with CI (*p* < 0.05): CI subjects = 21, HC subjects = 15.

## Supplementary information

Supplementary Material

Supplementary Figure 1

Supplementary Figure 2

Supplementary Figure 3
