# Supplementary material for: Astrocyte reactivity with late-onset cognitive impairment assessed in vivo using 11C-BU99008 PET and its relationship with amyloid load
Source: Mol Psychiatry. 2021 Jul 15;26(10):5848–55. doi: 10.1038/s41380-021-01193-z (PMC8758500; doi:10.1038/s41380-021-01193-z)
Supplement: Supplementary file 1 — Supplementary Material [file 41380_2021_1193_MOESM1_ESM.docx]

**Supplementary figure legends**

**Supplementary Figure 1: ^11^C-BU99008 regional IRF-120 in different cortical regions.**

Dot plot demonstrating the regional ^11^C-BU99008 IRF-120 in Aβ-positive patients (purple filled circle), Aβ-negative patients (purple open circle) and healthy controls (green triangle). “Brain” refers to the composite cortex, combining all the major cortical regions. * denotes p<0.05, uncorrected.

**Supplementary Figure 2: Correlation between ^11^C-BU99008 regional IRF-120 and 2TCM V_T._**

Scatter plots showing the correlation between ^11^C-BU99008 spectral IRF-120 parametric maps and 2TCM V_T_ in the four main lobes. Purple circles = healthy controls. Green circles = patients.

FL: Frontal Lobe; TL: Temporal Lobe; PL: Parietal Lobe; OL: Occipital Lobe

**Supplementary Figure 3: Single subject SPM analysis for ^11^C-BU99008 in all patients.**

Single subject SPM analysis of significant increased ^11^C-BU99008 uptake compared to all healthy controls, using a cluster threshold of p<0.05 and extent threshold of 50 voxels.

**Supplementary material**

1. **Material and methods: In vitro autoradiography**
   1. Compounds and radioligands

[^3^H]BU99008 was custom labelled (Moravek Biochemicals, USA) with a molar activity of 44.4 Ci/mmol. Unlabelled BU224, a high-affinity imidazoline 2 receptor binding ligand [56] used to determine nonspecific binding, was purchased from Tocris (UK). Unlabelled 6-OH-BTA-1 hydrochloride (PiB) and 4-{(E)-2-[4-(2-{2-[2-(18F)Fluoroethoxy]ethoxy}ethoxy)phenyl]vinyl}-N-methylaniline (Florbetaben) were purchased from ABX (Germany). All unlabelled compounds were dissolved in DMSO and added to the incubation buffer.

- 1. Tissue origin and sectioning

Human frontal cortex tissue from AD cases and control tissue was acquired through the London Neurodegenerative Diseases Brain Bank. All other human frontal cortex tissue was acquired through the Parkinson’s UK Brain Bank at Imperial College London.

From samples, 12 or 14 µm serial sections were cut on a cryostat (Leica CM1950, Germany), thaw-mounted onto Super Frost Plus slides (VWR, UK), briefly air dried and stored at -80°C until further processing.

- 1. In vitro autoradiography with [^3^H]BU99008

Sections were thawed at room temperature (RT) for 15 minutes, before being pre-incubated with assay buffer (50mmol/L Tris-HCl, 1.5 mM MgCl_2_, pH 7.4 (at RT) for a further 15 minutes. Total binding was assessed by incubating all samples for 60 minutes at RT in assay buffer containing 2-3.5 nMol/L [^3^H]BU99008 in a humidified chamber. Non-specific binding of [^3^H]BU99008 was assessed in the presence of 10 µMol/L BU224 for each individual in adjacent slides. In order to investigate potential binding of [^3^H]BU99008 to Aβ plaques, additional sections were incubated with [^3^H]BU99008, along with four increasing concentrations of unlabeled PiB/Florbetaben (10, 100, 1000 and 10000 nMol/L). Radioligand concentration was determined by liquid scintillation counting (Beckman Coulter LS6500 LSC).

Following incubation, slides were rinsed in Tris-HCl (1.5 mM MgCl_2_) and washed three times for 20 seconds on ice in 4°C wash buffer (50.0mmol/L Tris-HCl with 1.5mmol/L MgCl_2_) before a final 10 second wash in distilled water (dH2O) at 4°C. The slides were left to dry under an airstream, followed by further overnight desiccation under phosphorous pentoxide and were then exposed to phosphor-imaging plates (BAS-IP TR 2040, Fuji Film, Japan) together with Tritium radioactive standards (ART0123, B and C; American Radiolabelled Chemicals (ARC), USA) for at least 14 days in a lead lined storage unit.

- 1. Autoradiography data acquisition and analysis

Imaging plates were scanned using a storage phosphor imaging system (Typhoon FLA 7000; GE, UK). Quantity One 2D Analysis Software ® (Bio-Rad, UK) was used to manually draw regions of interest (ROIs) of the whole section (WB), grey matter (GM) and white matter (WM). Finally, the mean values of optical density per mm^2^ were converted to femtomole of tritiated radioligand per mg (fmol radioligand/mg) wet tissue equivalent (TE), by linear regression derived from the radioactive standards. A global background was subtracted as well as the non-specific binding to yield specific binding values. Final values were expressed as specific binding in fmol/mg TE.

- 1. Immunohistochemistry (IHC)

IHC was performed on tissue using the either the ABC method (Vectastain Elite, Vector Labs, UK) or the polymer-HRP Super Sensitive IHC Detection System (Biogenex, USA), with primary antibodies as shown in Table.

| **Antibody** | **Target** | **Dilution** | **Supplier** |
| --- | --- | --- | --- |
| Rabbit polyclonal anti-GFAP | GFAP | 1:10,000  0.29 µg/mL | DAKO, UK |
| Mouse pan anti-Aβ peptide, clone 6C3 | Aβ peptide | 1:1,000  1 µg/mL | Millipore, UK |

Tissue was thawed at room temperature for 15 minutes before post-fixation with 4% paraformaldehyde (PFA) on ice for 10 minutes. Sections were then washed twice in phosphate buffered saline (PBS) for 5 minutes, before being endogenous peroxidases were quenched using 1% H_2_O_2_ in PBS-Tween (PBS-T, 0.3% Tween) for 30 minutes. For ABC staining, sections were blocked with goat serum. Primary antibodies were applied and left to incubate overnight at 4^o^C. Primary antibodies were washed off before the incubation with the secondary antibody and staining with 3,3'-diaminobenzidine (DAB. Sections were then dehydrated in solutions of increasing concentrations of ethanol followed by xylene, before being mounted in DPX and coverslipped.

- 1. Immunohistochemistry data acquisition

Slides were scanned on an either an Axio Scan Z1 slide scanner at 20x magnification or a miscroscope (Eclipse E800, Nikon, UK) using a motorized stage at 10x.

- 1. Statistics

Differences between of ^3^H-BU99008 binding in AD cases and controls were tested using an unpaired t-test.
