# Supplementary figures and images for: Astrocyte reactivity with late-onset cognitive impairment assessed in vivo using 11C-BU99008 PET and its relationship with amyloid load
Source: Mol Psychiatry. 2021 Jul 15;26(10):5848–55. doi: 10.1038/s41380-021-01193-z (PMC8758500; doi:10.1038/s41380-021-01193-z)

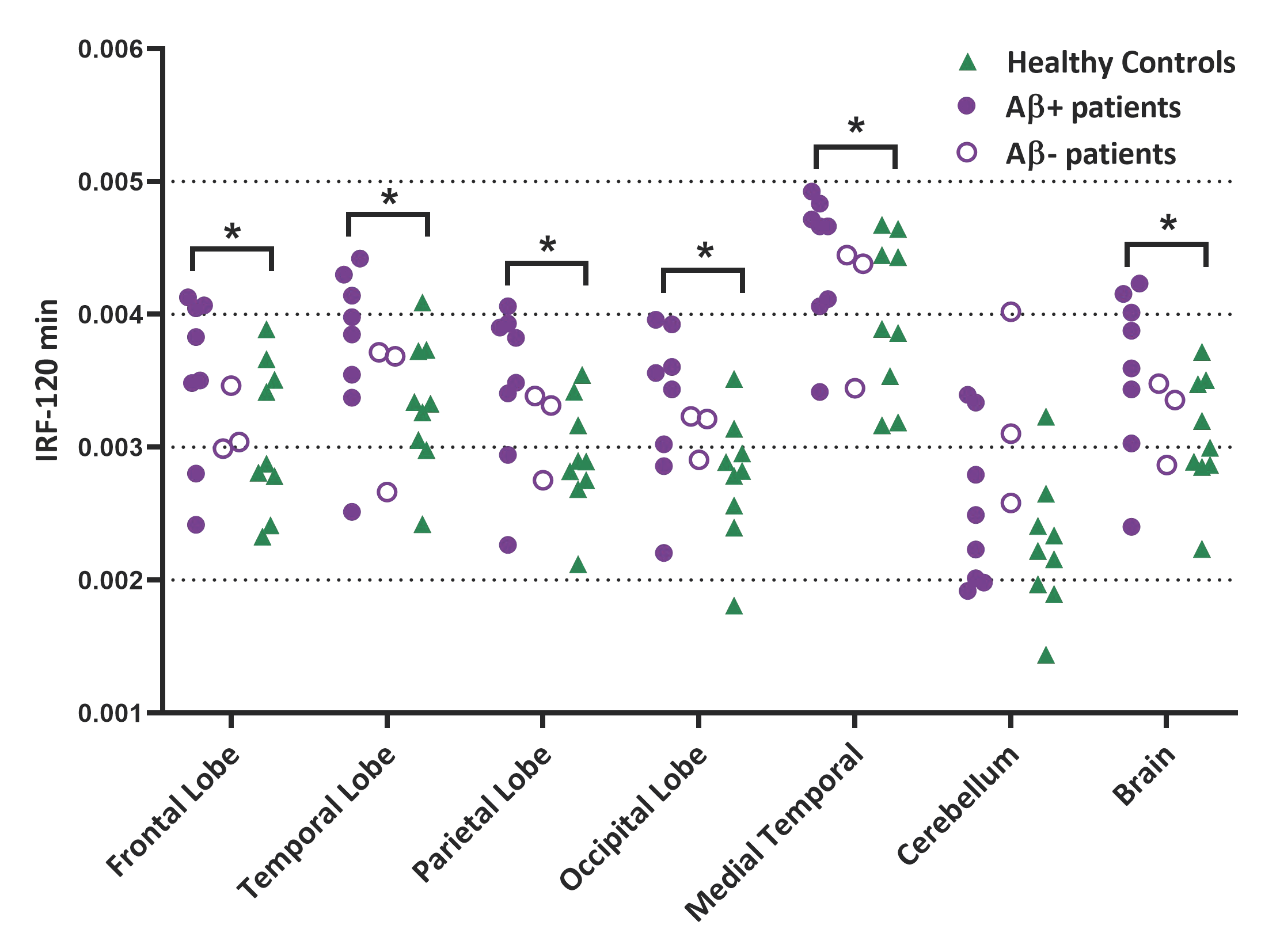

Supplement: Supplementary file 2 — Supplementary Figure 1 [file 41380_2021_1193_MOESM2_ESM.tiff]

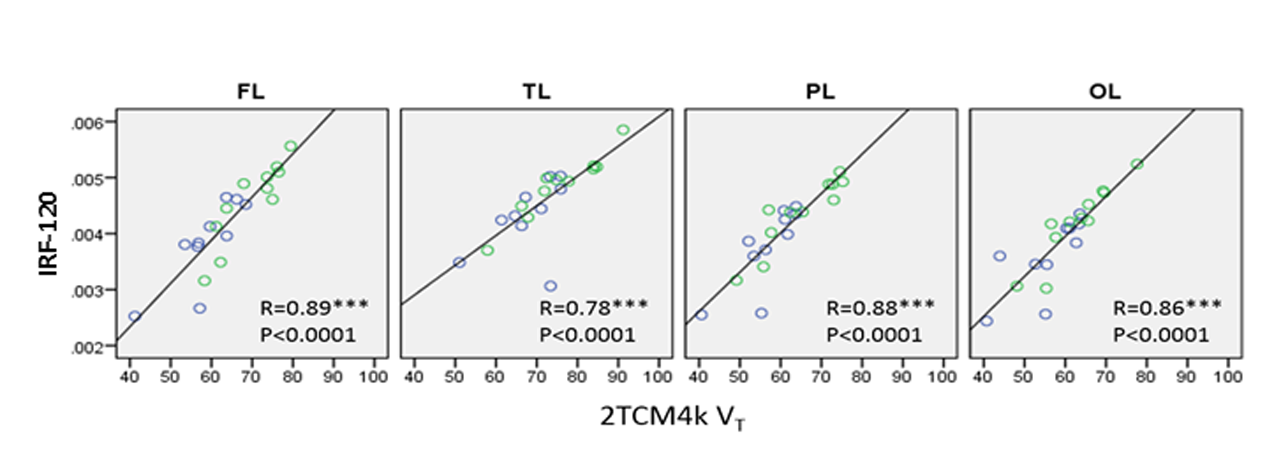

Supplement: Supplementary file 3 — Supplementary Figure 2 [file 41380_2021_1193_MOESM3_ESM.tif]

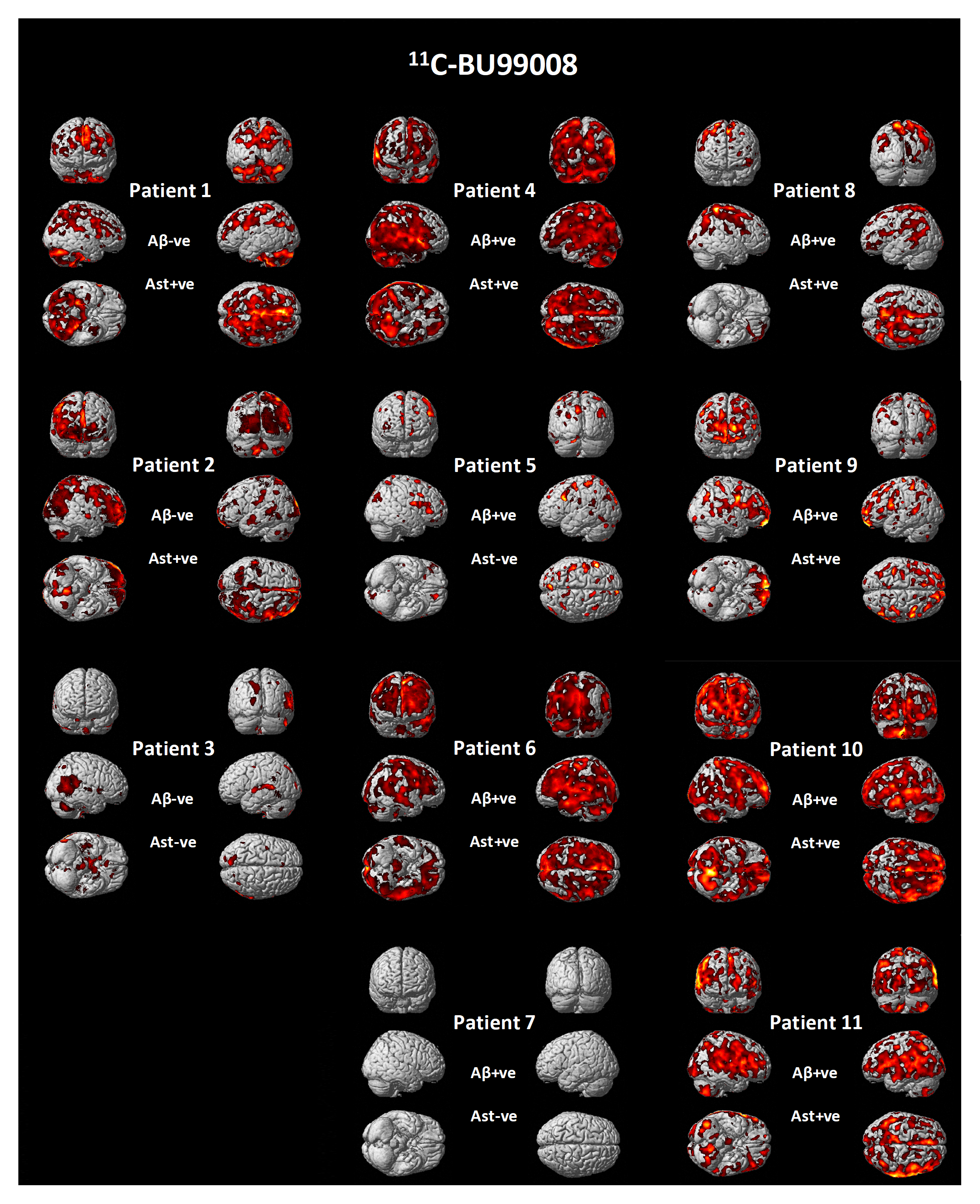

Supplement: Supplementary file 4 — Supplementary Figure 3 [file 41380_2021_1193_MOESM4_ESM.tif]
